# Coronary bypass grafting using crossclamp fibrillation does not result in reliable reperfusion of the myocardium when the crossclamp is intermittently released: a prospective cohort study

**DOI:** 10.1186/1749-8090-1-45

**Published:** 2006-11-21

**Authors:** Joel Dunning, Steven Hunter, Simon WH Kendall, John Wallis, W Andrew Owens

**Affiliations:** 1 Department of Cardiothoracic Surgery, James Cook University Hospital, Middlesbrough, UK

## Abstract

**Background:**

Cross-clamp fibrillation is a well established method of performing coronary grafting, but its clinical effect on the myocardium is unknown. We sought to measure these effects clinically using the Khuri Intramyocardial pH monitor.

**Methods:**

50 episodes of cross-clamping were recorded in 16 patients who underwent CABG with crossclamp-fibrillation. An Intramyocardial pH probe measured the level of acidosis in the anterior and posterior myocardium in real-time. The pH at the start and end of each period of cross-clamping was recorded.

**Results:**

It became very apparent that the pH of some patients recovered quickly while others entirely failed to recover. Thus the patients were split into 2 groups according to whether the pH recovered to above 6.8 after the first crossclamp-release (N = 8 in each group). Initial pH was 7.133 (range 6.974–7.239). After the first period of crossclamping the pH dropped to 6.381 (range 6.034–6.684). The pH in recoverers prior to the second XC application was 6.990(range 6.808–7.222) compared to only 6.455 (range 6.200–6.737) in patient's whose myocardium did not recover (P < 0.0005). This finding was repeated after the second XC release (mean pH 7.005 vs 6.537) and the third (mean pH 6.736 vs 6.376). However prior to separation from bypass the pH was close to the initial pH in both groups (7.062 vs 7.038).

**Conclusion:**

Crossclamp fibrillation does not result in reliable reperfusion of the myocardium between periods of crossclamping.

## Background

Cross Clamp fibrillationn is a well established technique for performing Coronary Bypass Grafting and it is routinely used by 15% of surgeons in the UK [1-3] Proponents of this technique report that this method subjects the heart to only short periods of ischaemia, with full reperfusion after every distal anastomosis. In addition the possibility that ischaemic preconditioning occurs as subsequent grafts are performed is proposed to further protect the myocardium [4-6]. It is commonly thought that ischaemic periods of up to 10–15 minutes are easily tolerated by the myocardium and clinical practise would suggest that cross-clamp fibrillation has identical outcomes to coronary bypass surgery using cardioplegia. However no study has yet been performed that has demonstrated adequate reperfusion between periods of cross clamp fibrillation and the phenomenon of ischaemic preconditioning has not yet been clinically demonstrated during cardiac surgery.

The Khuri Myocardial pH monitor has been recently developed that can for the first time provide real-time clinical analysis of the myocardial pH during cardiopulmonary bypass [7-10]. It has been well validated during cardiopulmonary bypass with cardioplegia in over 500 clinical cases and periods of low intramyocardial pH have been correlated with increased myocardial damage[11].

We sought to investigate the effect of cross-clamp fibrillation on the pH of the myocardium with the primary hypothesis that we may be able to define a 'safe-ischaemic time' for cross-clamping, and a secondary hypothesis that we may be able to measure ischaemic preconditioning in clinical practise for the first time.

## Methods

16 patients undergoing elective coronary bypass grafting using cross-clamp fibrillation had a Khuri intramyocardial pH monitor placed intra-operatively. The monitor consists of two probes. The first (anterior) probe is placed prior to cardiopulmonary bypass into the left ventricle, lateral to the LAD, directed obliquely into the anterior ventricular septum. The second probe is placed lateral to the posterior descending artery, again in an oblique fashion so that the tip rests in the posterior portion of the ventricular septum. Continuous pH monitoring is then performed with pH measurements recorded every 10 seconds. Cardiopulmonary bypass was performed at 37 degrees centigrade, but the pH probe also contains a temperature probe and provides a pH value corrected to normothermia.

Operative technique was not modified in any way for this study. The PDA or RCA was first grafted with the cross clamp applied for the distal anastomosis and released as the proximal anastomosis is formed. Lateral wall grafts were then performed, with the LAD being the last graft to be placed. The maximal pH recovery was determined as the pH just before the subsequent cross clamp placement. The posterior probe was removed prior to separation from bypass but the anterior probe remained in place until separation from bypass and a constant final pH reading was achieved.

### Statistical analysis

The pH recordings were analysed and the pH recordings prior to, 5 minutes into and after crossclamping were recorded. The average values of the anterior and the posterior probe recordings were taken. In addition the maximum pH recovery after crossclamp removal was documented (which was recorded only by the anterior probe). 2 groups were retrospectively defined according to whether the pH recovered to above 6.8. The cut-off point of 6.8 was determined purely by an observational assessment and the conclusion that patients whose pH did not recover to above this level seemed to be different to those that did. The 2 groups were created simply to highlight this observational difference. The pH values were compared between these groups using the unpaired Student t-test, after normality was established using the Kolmogorov-Smirnov test.

## Results

**Figure 1:**
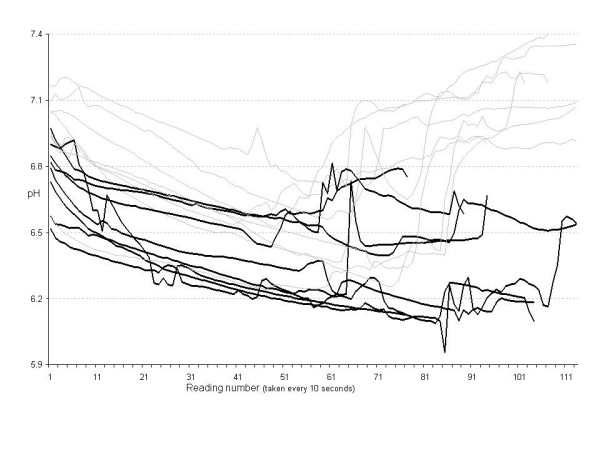
pH recordings from application of crossclamp, though release, until application of second cross clamp, allowing maximal time for recovery. The traces that recovered are represented by the light lines, and the traces that failed to recover are represented by the heavy black lines.

**Figure 2:**
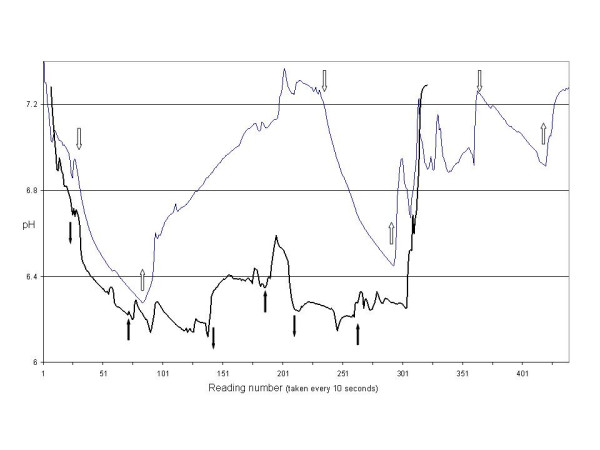
An example of a patient with successful reperfusion (Top light line) and a patient with unsuccessful reperfusion (lower heavy line). The arrows represent application and release of the crossclamp. Of note the patient that did not have recovery received a RIMA graft as the second anastomosis.

20 patients were entered into the study but 3 patients had malpositioning of the pH probe and puncture of the left ventricle causing misreading, and in a 4^th ^patient the probe was opened but not used. The remaining 16 patients had 50 periods of crossclamping to fashion 47 distal anastomoses. The demographics of these patients are shown in table 1. When the traces were analysed, it became clear that the pH recovered well on release of the crossclamp for some patients, but in other patients there was a complete failure to recover (See figures 1 and 2). We therefore split the data into two groups as well as analysing the traces as a whole. The full results of the analysis are shown in table 2.

**Table 1:** Demographics of the patient cohort

	**Total (N = 16)**	**Recoverers (N = 8)**	**Non Recoverers (N = 8)**
**Age**	**67 **(SD 9)	**66 **(SD 2)	**67 **SD(4)
**Male**	**15**	**7**	**8**
**Diabetes (on insulin)**	**1**	**0**	**1**
**Ex/current smokers**	**12**	**6**	**6**
**BMI**	**28 **(SD 3)	**29 **(SD 1)	**27 **(SD 1)
**EF**	**63 **(SD 14)	**61 **(SD 7)	**65 **(SD 4)
**Prev MI**	**7**	**5**	**2**
**LMS disease**	**9**	**5**	**4**
**Triple vessel disease**	**14**	**6**	**8**
**Euroscore**	**2.6 **(SD 1.7)	**2.6 **(SD 0.7)	**2.5 **(SD 0.5)
			
**INTRAOPERATIVE DATA**			
**CABG × 2**	**2**	**2**	**0**
**CABG × 3**	**13**	**6**	**7**
**CABG × 4**	**1**	**0**	**1**
**BIMA usage**	**4**	**1**	**3**
**Mean single XC time**	**9 (SD 2) range 4–15 mins**	**8.6 (SD 0.3) range 5–12 mins**	**9.5 (SD 0.5) range 4–15 mins**
**XC time**	**29 **(SD 7)	**28 **(SD 3)	**30 **(SD 2)
**Bypass time**	**68 **(SD 16)	**69 **(SD 7)	**67 **(SD 4)

**Table 2:** Results of pH analysis with Khuri Intramyocardial pH probe

	Total Ph	Recoverers	non recoverers	p value
Initial pH	7.133 (SD 0.0635) range 6.974–7.239	7.132 (SD 0.0121)	7.134 (SD 0.0305)	0.958
pH after 1st XC	6.381 (SD 0.165) range 6.034–6.684	6.445 (SD 0.126)	6.317 (SD 0.183)	0.124
pH Recovery after 1^st ^XC	6.723 (SD 0.324) range 6.200–7.222	6.990 (SD 0.561) range 6.808–7.222	6.455 (SD 0.067) range 6.2000–6.737.	<0.0005
pH after 2^nd ^XC	6.495 (SD 0.192) range 6.118 – 6.820	6.610 (SD 0.131)	6.381 (SD 0.180)	0.012
Ph Recovery after 2^nd ^XC	6.769 (SD 0.303) range 6.210 – 7.159	7.005 (SD 0.1193)	6.537 (SD 0.240)	<0.0005
pH after 3^rd ^XC	6.530 (SD 0.267) range 6.19 to 6.89	6.736 (SD 0.159)	6.376 (SD 0.224)	0.006
pH recovery after 3^rd ^XC	6.919 (SD 0.304) range 6.23 to 7.28	7.081 (SD 0.0838)	6.798 (SD 0.356)	0.084
Final pH	7.050 (SD 0.188) range 6.55–7.33.	7.062 (SD 0.0945)	7.038 (SD 0.258)	0.810
pH drop for each period of cross clamping				
Drop in 5 mins 1^st ^XC		**0.355 (SD 0.0690)**	0.320 (SD 0.165)	
Drop in 5 mins 2^nd ^XC		**0.186 (SD 0.175)**	0.0742 (SD 0.0708)	
Drop in 5 mins 3^rd ^XC		**0.152 (SD 0.0696)**	0.103 (SD 0.119)	
	P value	**0.009**		

The initial pH was 7.133 (range 6.974–7.239) for the group as a whole. On cross-clamping the pH reduced, with mean pH after the first cross-clamp removal being 6.381 (SD 0.165 and range 6.034–6.684). The recovery after cross clamp removal was highly inconsistent. The mean pH at the end of the first period of cross clamp release was 6.723 (SD 0.324), but in patients we termed as non-recoverers the mean pH rose to only 6.455 (SD 0.067) compared to recoverers whose pH rose to 6.990 (SD 0.561). These findings were repeated for subsequent applications of the cross-clamp. However at the end of the period of pH measurement (Once off bypass) there was no significant difference in myocardial pH between these two groups [7.062 (SD 0.0945) in recoverers and 7.038 (SD 0.258) in the non-recoverers (p = 0.810)].

We further subanalysed the rate of descent of the pH in the 8 patients who did have acceptable recovery of the pH on cross clamp removal. We found that the rate of descent after the first cross clamp was 0.355 in 5 minutes, the rate of descent after the second cross clamp was 0.186 in 5 minutes and the rate of descent after the third cross clamp was 0.0152 in 5 minutes. This was a significant reduction in the rate of descent in pH as the number of periods of cross-clamping increased (p = 0.009). The clinical outcomes for the 16 patients are shown in table 3.

**Table 3:** Post-operative results of patients undergoing pH analysis

	**Total (N = 16)**	**Recoverers (N = 8)**	**Non Recoverers (N = 8)**
Mean CICU Stay (range)	1.25 (1–3)	1.13 (1–2)	1.38 (1–3)
Mean Hospital stay (range)	7.81 (4–17)	7.38 (4–14)	8.25 (4–17)
Inotrope usage	0	0	0
Atrial Fibrillation	3	1	2
Resternotomy	1	1	0

## Discussion

To our knowledge this is the first study to investigate the level of ischaemia found in the myocardium during coronary arterial bypass grafting using the technique of cross-clamp fibrillation. We have documented for the steady reduction in pH during this technique, which reaches pH levels as low as 6.03, and on average the pH dropped to 6.38 prior to first cross clamp removal.

We have also documented for the first time 8 patients whose intramyocardial pH did not recover once the cross clamp was removed. We hypothesise that the reason for this lack of recovery is that despite removal of the cross-clamp, blood did not perfuse the native coronary arteries (or the graft after second cross clamp removal). The reasons for this lack of perfusion require further research but possibilities would include the much lower aortic root pressure than the physiological situation (or the pressure that cardioplegia is given in patients undergoing cardiopelgic techniques) and changes in the resistance of heavily diseased vessels that may now be twisted or compressed.

One further interesting preliminary finding is that in those patients who do undergo ischaemia and then recovery, the rate of descent of the pH reduced significantly on subsequent cross clamp applications. To our knowledge this is the first demonstration in the clinical situation that ischaemic preconditioning has been shown. This clinical demonstration confirms a large number of animal studies performed up to 20 years ago that clearly show the existence of ischaemic preconditioning [12-15].

Our study has many weaknesses due to its preliminary nature. The splitting of our patients into 2 groups was entirely retrospective in nature and was done in order to understand further the results we were seeing for the first time. However while we felt that the two groups behaved very differently, there may in fact be a spectrum of patients rather than two distinct groups. This requires further patients to undergo intramyocardial pH monitoring during cross-clamp fibrillation. Secondly our initial report of ischaemic preconditioning is based on only 8 patients and this requires many more patients to establish that this is a physiological phenomenon in everyday clinical practise.

It has been clearly and repeatedly demonstrated in well-conducted prospective randomized trials that the technique of cross clamp fibrillation is equivalent to both crystalloid and blood cardioplegia in terms of clinical outcome and biochemical markers of myocardial damage [16-22]. In 1998 Liu et al[17] compared the results of 1,345 patients who had cross-clamp fibrillation with 578 patients who had coronary arterial bypass grafts with cold crystalloid cardioplegia at one institution during the same time period. The groups had identical mortality, stroke rates, ICU stay, haemofiltration rates and resternotomy rates. Alhan et al[20] compared 128 patients having CABG with cold crystalloid cardioplegia with 271 patients having CABG with crossclamp fibrillation, and found no differences in clinical outcomes, including mortality, IABP use, inotropic requirements and myocardial infarction. Gerola et al[22] performed a prospective randomized trial of 60 patients randomized to either aspartate-glutamate enriched warm blood cardioplegia or cross clamp fibrillation. Again there were no differences in mortality, cardiac index, or myocardial infarction rates. Finally, in a prospective randomized trial, Musumeci et al[18] randomized 43 patients to cross clamp fibrillation and 48 to cold blood cardioplegia. Levels of CK. MB, S-100B, Troponin I and T, LV function, ECG changes and number of cerebral micro-emboli were either similar between the two groups or superior in the crossclamp fibrillation group.

Thus clinically and biochemically cross clamp fibrillation is indistinguishable from cardioplegic techniques. In contrast Khuri et al have performed over 500 measurements of intamyocardial pH during cardiac surgery using cardioplegia. He has correlated a pH of below 6.87 at the end of bypass with an increase in inotrope requirement[7] and a pH of below 6.34 during crossclamping with an increase in long term mortality[8,9]. In addition he has found that the level of apoptosis in samples of myocardium exposed to a pH of 6.34 was three times higher than tissue at normal pH[11].

We have established that the pH of the myocardium routinely falls to very low levels during cross clamp fibrillation. However repeated studies have shown equivalence between cross clamp fibrillation and cardioplegic techniques. Thus it is possible that Khuri's findings of poorer short and long term outcomes with lower pH levels in patients having surgery using cardioplegia would not extrapolate to patients having periods of profound myocardial acidosis during cross clamp fibrillation.

Our preliminary study cannot answer whether ischaemic preconditioning accounts for the heart's ability to sustain this profound ischaemia during cross clamp fibrillation and indeed our finding of several patients who had sustained ischaemia suggest that the mechanisms by which the myocardium is protected from apoptosis remain unknown.

We suggest that further studies of patients undergoing crossclamp fibrillation are performed in order to further elucidate the factors that allow adequate reperfusion and also to investigate the clinical phenomenon of ischaemic preconditioning. Furthermore we were unable to determine a 'safe-period' for crossclamp application due to the intermittent nature of reperfusion. Once further studies are performed to elucidate the conditions for reliable reperfusion, a 'safe-ischaemic time' may become possible to determine.

## Conclusion

Crossclamp fibrillation does not result in reliable reperfusion of the myocardium between periods of crossclamping.

## Abbreviations

LIMA: Left Internal Mammary Artery.

BIMA: Bilateral Internal Mammary Arteries

CK-MB: Creatinine Kinase-Myocardial Band

SD: Standard Deviation

PDA: Posterior Descending Artery

RCA: Right Coronary Artery

LAD: Left Anterior Descending Artery

LMS: Left Main Stem Coronary Artery

UK: United Kingdom

CABG: Coronary Arterial Bypass Grafts

BMI: Body Mass Index

XC: Cross Clamp

EF: Ejection Fraction

MI: Myocardial Infarction

CICU: Cardiothoracic Intensive Care Unit

## Competing interests

The author(s) declare that they have no competing interests.
